# Geographical differences in perinatal health and child welfare in the Netherlands: rationale for the healthy pregnancy 4 all-2 program

**DOI:** 10.1186/s12884-017-1425-2

**Published:** 2017-08-01

**Authors:** Adja J. M. Waelput, Meertien K. Sijpkens, Jacqueline Lagendijk, Minke R. C. van Minde, Hein Raat, Hiske E. Ernst-Smelt, Marlou L. A. de Kroon, Ageeth N. Rosman, Jasper V. Been, Loes C. M. Bertens, Eric A. P. Steegers

**Affiliations:** 1 Department of Obstetrics and Gynaecology, Division of Obstetrics and Prenatal Medicine, Erasmus Medical Centre Rotterdam, Postbus 2040, 3000 CA Rotterdam, the Netherlands; 2 Department of Public Health, Erasmus Medical Centre Rotterdam, Postbus 2040, 3000 CA Rotterdam, the Netherlands; 3 Department of Paediatrics, Division of Neonatology, Erasmus Medical Centre Rotterdam, Postbus 2040, 3000 CA Rotterdam, the Netherlands

**Keywords:** Perinatal health inequalities, Perinatal mortality, Perinatal morbidity, Child welfare, Risk assessment, Child health care, Deprived neighbourhoods, Public health, Interconception care, Maternity care

## Abstract

**Background:**

Geographical inequalities in perinatal health and child welfare require attention. To improve the identification, and care, of mothers and young children at risk of adverse health outcomes, the HP4All-2 program was developed. The program consists of three studies, focusing on creating a continuum for risk selection and tailored care pathways from preconception and antenatal care towards 1) postpartum care, 2) early childhood care, as well as 3) interconception care. The program has been implemented in ten municipalities in the Netherlands, aiming to target communities with a relatively disadvantageous position with regard to perinatal and child health outcomes. To delineate the position of the ten participating municipalities, we present municipal and regional differences in the prevalence of perinatal mortality, perinatal morbidity, children living in deprived neighbourhoods, and children living in families on welfare.

**Methods:**

Data on all singleton births in the Netherlands between 2009 and 2014 were analysed for the prevalence of perinatal mortality and morbidity. In addition, national data on children living in deprived neighbourhoods and children living in families on welfare between 2009 and 2012 were analysed. The prevalence of these outcomes were calculated and ranked for 62 geographical areas, the 50 largest municipalities and the 12 provinces, to determine the position of the municipalities that participate in HP4All-2.

**Results:**

Considerable geographical differences were present for all four outcomes. The municipalities that participate in HP4All-2 are among the 25 municipalities with the highest prevalence of perinatal mortality, perinatal morbidity, children living in deprived neighbourhoods, or children in families on welfare.

**Conclusion:**

This study illustrates geographical differences in perinatal health and/or child welfare outcomes and demonstrates that the HP4All-2 program targets municipalities with a relative unfavourable position. By targeting these municipalities, the program is expected to contribute most to improving the care for young children and their mothers at risk, and hence to reducing their risks and health inequalities.

**Electronic supplementary material:**

The online version of this article (doi:10.1186/s12884-017-1425-2) contains supplementary material, which is available to authorized users.

## Background

Suboptimal health before birth and in early life has long term consequences for children, their families, and next generations [1]. Moreover, substantial (perinatal) health inequalities are present between, and within, high-income countries. In the Netherlands, perinatal mortality rates are higher than in many other European countries [2], and these rates differ widely between regions and even between neighbourhoods [3–5].

Living in a deprived region is acknowledged as an important risk factor for adverse birth outcomes, such as preterm birth and small-for-gestational age birth [3, 6, 7]. In deprived regions the prevalence of risk factors, single or in combination, is higher than in non-deprived regions [8, 9]. Not only medical risks, but also non-medical risk factors are involved, often related to poverty, such as low socioeconomic status, substance abuse including smoking, and psychological distress [9].

Since 2008, in response to the awareness about the high prevalence of adverse perinatal outcomes in the Netherlands, much effort has been invested into improving perinatal health [10]. This has led to research and policy programs that aim to increase attention for risk assessment and risk reduction before and during pregnancy. One such program, ‘Ready for a Baby’ (2008–2012), was initiated with the aim to improve perinatal health in Rotterdam, the second largest city in the Netherlands, especially in its deprived neighbourhoods [11, 12]. Strengthening of the inter-professional collaboration between curative and the public health professionals and reaching-out to a more vulnerable population, consisting of low-educated and/or immigrant groups, were the stepping stones to reach this goal.

In 2011, building on the insights of the ‘Ready for a Baby’ program, we launched the Healthy Pregnancy 4 All (HP4All-1) program in 14 municipalities that had higher rates of adverse perinatal outcomes than the national average [4]. The HP4All-1 program focused on: a) the implementation of preconception care via different recruitment strategies, and b) the introduction of systematic antenatal risk assessment (considering both medical and non-medical risk factors) with the antenatal Rotterdam Reproductive Risk Reduction (R4U) scorecard, followed by tailored multidisciplinary care pathways [13, 14]. Again, optimal linkage between the curative and the public health domain was sought on preconception, prenatal and perinatal care.

Since 2014, this approach has been extended to cover postpartum care, early childhood care and interconception care in the Healthy Pregnancy for All 2 (HP4All-2) program.

### HP4All-2 program

The HP4All-2 program focuses on creating a continuum of risk selection, followed by tailored (multidisciplinary) care pathways, from the preconception and prenatal period towards the postpartum and early childhood period. The rationale for this focus is that certain risk factors before and during pregnancy, such as neighbourhoods and individual social characteristics, often continue to exist after delivery, affecting both maternal and offspring health [6, 15]. Moreover, perinatal health status in itself is an important determinant of child health and health in later life [1]. For example, high birth weight is positively associated with childhood overweight and low birth weight is negatively associated with developmental outcomes [16, 17]. To translate this knowledge into practice, comprehensive care beyond the boundaries of the separate social and medical domains of care is needed in the preconception, prenatal, postpartum and early childhood period [18].

Therefore, HP4All-2 aims to introduce integrated, risk-guided care, beyond separate domains of antenatal care, maternity care and Preventive Child Health Care (PCHC). In the Netherlands, professional maternity care is provided at home by maternity care assistants, who have completed a specialisation of ‘personal health care assistant’ at the level of secondary vocational education and are being supervised by community midwives [19]. PCHC organizations promote children’s health up to the age of 19 years by providing immunisations, monitoring growth and development, offering health advice, and referring to specialised care if needed [20, 21].

Maternity care and PCHC are used as the main settings for three risk assessment interventions that are studied within the HP4All-2 program. These three intervention studies are being implemented in ten municipalities that agreed to participate in one or more of the studies (Table 1).

**Table 1:** An overview of the participation of municipalities in the HP4All-2 program, and its studies

Municipality	Maternity care study^a^	PCHC study^b^	Interconception care study^c^
Amsterdam^d^		X	X
Rotterdam^d^	X	X	X
Den Haag^d^			X
Utrecht^d^	X		
Tilburg^d^			X
Groningen^d^	X		X
Almere^d^	X		X
Arnhem	X		
Dordrecht		X	
Schiedam^d^	X		X

^a^ Structured risk assessment during pregnancy and customised maternity care study

^b^ Optimizing postnatal risk assessment in PCHC study

^c^ Interconception care study through PCHC

^d^ selection based on their participation in earlier programs (‘Ready for a Baby’ or HP4All-1)

- **Study 1:** Structured risk assessment during pregnancy and customised maternity care

*Aim* This study aims to timely plan customised maternity care to the individual needs of women at high risk for adverse pregnancy and child outcomes.

*Rationale* Previous research indicates that high risk women benefit more from intensive postpartum care than women with low risks [22, 23]. This yields the need for a structured risk assessment during pregnancy in conjunction with custom fit maternity care.

*Study Design* This study is a cluster randomised controlled trial in six municipalities in the Netherlands. Within a municipality, two clusters are formed in the same geographical area; one intervention and one control cluster. Two municipalities were merged together to account for enough participants, resulting in a total number of 10 clusters. A cluster may consist of one or more maternity care organisations. The intervention under study is a systematic risk assessment during pregnancy of medical and non-medical risk factors for adverse maternal and child outcomes, in conjunction with client-tailored care during pregnancy and the postpartum period. In the control clusters this systematic risk assessment is introduced during pregnancy as well, yet is followed by conventional maternity care during pregnancy and in the postpartum period. All pregnant women cared for by participating maternity care organisations, who have a scheduled home visit during pregnancy, are invited to take part in the trial.

*Outcomes* Primary outcome is maternal empowerment assessed between day 6 and 14 postpartum. Secondary outcome measures include maternal health outcomes, maternal health behaviour and health care utilisation in the first months postpartum. In addition, we will assess the determinants of successful implementation by questionnaires addressed to managers of maternity care organisations and to maternity care assistants.

- **Study 2:** Optimising postnatal risk assessment in Preventive Child Health Care

*Aim* This study aims to identify and reduce the risk of growth and developmental problems in children before the age of 18 months, during their postnatal visits to the PCHC centre.

*Rationale* Within PCHC centres, care is provided to all children and families free of charge, with population coverage of 95% during the first year of life. Therefore, it seems to be the ideal setting for early risk screening and indicating appropriate care for vulnerable families at risk of adverse child health outcomes. To ensure structured risk assessment, the ‘postnatal R4U’ has been developed (comparable to the ‘antenatal R4U’ [13]). This risk assessment instrument scores both medical and non-medical risk factors and combines information already documented by the PCHC, obstetric data and newly screened items. All items of the ‘postnatal R4U’ are based on an extensive literature search and expert consultations by focus group interviews. In summary, the items were categorised into six domains: the social [24–26], ethnicity [17, 27], care status [28], lifestyle [29–31], obstetric [32, 33] and medical domains [34, 35].

*Study design* In this prospective cohort study, the ‘postnatal R4U’ is introduced in the participating PCHC centres in three municipalities. All children aged zero to 8 weeks old will be assessed with this instrument and, in case of detected risks, integrated care pathways will be offered to reduce the detected risks. A historical control group of children in the same four-digit postal code area will be constructed for comparison of the study outcomes.

*Outcomes* Primary outcomes are growth problems (defined as overweight, obesity and catch-up growth) and developmental problems in children until the age of 18 months. Developmental problems will be assessed using the ‘Van Wiechen Scheme’, a Dutch instrument for monitoring motor, language, cognitive and psychosocial development which is routinely applied from birth onward at visits to the PCHC centre [36].

- **Study 3:** Interconception care through Preventive Child Health Care

*Aim* This study aims to implement and evaluate interconception care in PCHC centres.

*Rationale* Interconception care, also referred to as preconception care between pregnancies, aims to facilitate optimal preparation for pregnancy and minimise risk factors for an adverse pregnancy outcome. Delivery of interconception care is still uncommon [37]. A valuable opportunity to deliver interconception care can be through PCHC centres, since almost all parents and their young children visit PCHC centres regularly for routine well-child visits [38].

*Study Design* In this prospective cohort study, interconception care is implemented in participating PCHC centres in seven municipalities. PCHC professionals are instructed to inform women about the possibility of an interconception care consultation in case of a (future) pregnancy wish. They discuss this possibility with women who attend for a routine visit at their child’s age of 6 months. Subsequently, women can make an appointment for a separate interconception care consultation. In three municipalities women are offered this consultation by the PCHC centre, in the other four municipalities they are referred to local midwives or general practitioners. Decisions on which approach was applied, were made in mutual agreement with stakeholders within the municipalities.

Professionals are requested to record each time they discuss the possibility of an interconception care consultation with women, as well as when they provide the actual consultation.

*Outcomes* Primary outcome is the effectiveness of the implementation of interconception care in PCHC, measured as the proportion of eligible women who were informed about an interconception care consultation. Secondary outcomes include determinants of the implementation, effectiveness and utilisation of interconception care, studied by surveying women with a (future) pregnancy wish and PCHC professionals.

The HP4All-2 program is currently implementing these studies, aiming to target municipalities with a relatively disadvantageous position on perinatal and child health outcomes. In 2014 we presented data on regional perinatal health outcomes in the Netherlands during the period 2000–2008, based on which municipalities were invited to participate in the HP4All-1 program [4]. To delineate the recent position of the ten currently participating municipalities relative to other regions in the Netherlands, we now present the municipal and regional prevalence of perinatal mortality and morbidity over the period 2009–2014. Additionally, given the focus of the HP4All-2 program on postnatal care in continuum with antenatal care, proxies for socioeconomic risk factors for adverse child health are included in our analyses, being the prevalence of children living in deprived neighbourhoods and of children living in families on welfare over the period 2009–2012.

## Methods

### Data sources

National data on all singleton births from 22 weeks of gestation onwards between 2009 and 2014 were obtained from Perined (www.perined.nl) in April 2016. Perined contains information on more than 97% of all pregnancies in the Netherlands. Pregnancy, delivery, and neonatal data are routinely collected by midwives, gynaecologists and paediatricians [39]. A detailed description of the linkage procedures can be found on the Perined website (www.perined.nl).

Small area-level data on the proportion of children living in deprived neighbourhoods and of children living in families on welfare between 2009 and 2012, were provided by the ‘Defense for Children’ (www.defenseforchildren.nl), a Dutch non-governmental Coalition for Children’s Rights. This coalition monitors data on child well-being, based on ‘Kid’s Count’, a method used in the USA [40, 41]. The data of both outcomes applied to the age group 0 up to and including 17 years, and were available per four-digit postal code per year. Details on the definitions of these outcomes are available at the website (www.defenseforchildren.nl).

Data from Statistics Netherlands (CBS, www.CBS.nl) were used to identify the 50 largest municipalities of the Netherlands, based on the number of inhabitants in January 2015 (all above 70,000 inhabitants).

The four-digit postal code from the Perined database was used to assign each pregnancy to one of these 50 municipalities or to one of the 12 provinces (excluding the 50 previously selected municipalities). In the same way, the data on children living in deprived neighbourhoods and living in families on welfare were assigned to one of these 62 geographical areas.

Data on socioeconomic status (SES) were based on an area-level SES indicator by four-digit postal code, constructed by the Netherlands Institute for Social Research (SCP, www.scp.nl) over the year 2014. The SES indicator had been composed by a principal component analysis of the following items: 1) mean annual income per household, 2) percentage of households with low income, 3) percentage of households with low education and (4) percentage of unemployed inhabitants [42].

The SES data were linked to the data on pregnancies using the four-digit postal code.

### Outcomes

*Perinatal mortality*: was defined as death occurring between 22 weeks of gestational age and 7 days after birth. This determinant includes foetal mortality, intrapartum mortality and early neonatal mortality.

*BIG2*: was defined as small for gestational age (SGA) and/or preterm birth. SGA was defined as a birth weight below the 10th centile adjusted for ethnicity, parity, gestational age, and gender [43]. Preterm birth was defined as any birth occurring before 37 + 0 weeks of gestational age.

*Proportion children living in deprived neighbourhoods*: was defined as the number of children, in the age group zero up to and including 17 years, living in deprived neighbourhoods per municipality, divided by the total number of children of that age living in that municipality.

*Proportion children living in families on welfare*: was defined as the number of children in the age group 0–17 years, living in families on welfare per municipality, divided by the total number of children of that age living in that municipality.

### Determinants

*Ethnicity*: the mothers’ ethnicities were categorised into Western and non-Western. Western consisted of Dutch and other European nationalities. Non-western consisted of all other (i.e. non-European) ethnicities.

*Socioeconomic status*: the SES-scores where categorised into three groups: ‘Low’, a SES-score below the 20th centile; ‘Medium’, from the 20th up to and including the 80th centile; and ‘High’, above the 80th centile.

*Parity*: the mothers’ parity was dichotomised into 2 categories: ‘Primiparity’ including all first time pregnancies; and ‘Multiparity’, including all subsequent pregnancies.

### Missing data

The amount of missing data varied across determinants and ranged between 0.01% (parity) and 1.6% (ethnicity). In the data provided, there were no missing data on perinatal mortality, BIG2, children living in deprived neighbourhoods, and children living in families on welfare. Each determinant was assessed on unlikely or contradictory values. These unlikely values were found in the determinants ‘age of the mother’ (values below 10 years of age), and ‘postal code’ (if area code was officially labelled as uninhabited). Unlikely values were considered as missing data. Missing data were not imputed, as the determinants containing missing data were only used to describe the population and there were no missing data for each of the outcomes.

### Statistical analyses

Firstly demographic characteristics (i.e. age, ethnicity, parity, and SES) of all singleton births, as well as perinatal outcomes and child welfare outcomes were tabulated according to whether these occurred in one of the four largest cities of the Netherlands (Amsterdam, Rotterdam, The Hague, and Utrecht (the G4)), in analogy to Denktaş et al. [4].

Secondly, to delineate the recent position of the participating HP4All-2 municipalities relative to other regions in the Netherlands, each birth was assigned to one of the 62 selected geographical areas (50 largest municipalities and 12 provinces), and the geographical prevalence (per 1000 births) of perinatal mortality, BIG2, children living in deprived neighbourhoods, and children living in families on welfare was calculated. Maps were constructed to graphically illustrate these distributions.

Thirdly, the calculated prevalence per geographical area for all four outcomes was used to construct a ranking of the geographical areas. For each outcome, rank 1 was assigned to the geographical area with the highest prevalence and rank 62 to the area with the lowest prevalence.

Finally, the prevalence of known socio-demographic risk factors for adverse perinatal outcomes for which we had data (i.e. age of mother below 20, non-Western ethnicity, primiparity, and low SES) were tabulated against the 62 geographical areas.

The analyses were based on non-blinded data, since we based our analyses on national registry data independent of the HP4All-2 program. Analyses were performed using R version 3.2.3 (2016, The R Foundation for Statistical Computing) and ArcGIS 9.3, a geographical information system (release NL-16m07).

## Results

Of the 1,027,556 births in the Netherlands registered with Perined over the period 2009–2014, 1,009,687 (98%) were singleton pregnancies, and used for the analyses. In Table 2 characteristics of these pregnancies are tabulated by whether women lived in one of the four largest cities or in the rest of the Netherlands (The Netherlands minus the four largest cities). Regarding the total number of the births in the Netherlands, the median age of the mother was 30 years (interquartile range: 27–40) and the mothers’ ethnicity was predominantly Western (86%).The overall perinatal mortality over the period between 2009 and 2014 was 7.8 per 1000 births. Perinatal morbidity, represented by BIG2, was 142 per 1000 births.

**Table 2:** Population characteristics of the singleton births between 2009 and 2014 and child welfare outcomes between 2009 and 2012, stratified by location in the four largest cities (G4) or in the rest of the Netherlands

	G4-cities	The Netherlands minus G4-cities	Total
Singleton births	174,989	834,698	1,009,687
*Parity*			
Primiparous	49.0	45.2	45.9
Multiparous	51.0	54.8	54.1
*Ethnicity*			
Western	65.1	89.7	85.5
Non- Western	34.9	10.3	14.5
*Maternal age*			
<20 years	1.6	1.2	1.2
20–24 years	10.5	10.1	10.2
25–29 years	25.1	31.7	30.6
30–34 years	37.1	37.1	37.1
≥35 years	25.7	19.8	20.9
*Socioeconomic status score*			
Low (< p20)	39.5	16.0	20.1
Middle (p20 – p80)	32.3	65.7	59.9
High (> p80)	28.2	18.3	20.0
*Perinatal outcomes*			
Congenital anomalies	2.3	2.7	2.7
Preterm birth	6.2	6.1	6.1
Small for gestational age	10.2	8.3	8.7
Apgar score < 7 (5 min after birth)	2.3	1.9	1.9
Any BIG2^a^	15.7	13.9	14.2
Fetal mortality	0.32	0.30	0.30
Intrapartum mortality	0.20	0.17	0.18
Early neonatal mortality	0.34	0.29	0.30
Perinatal mortality^b^	0.86	0.76	0.78
Children 0–17 years (4 years^c^)	1,692,985	12,339,094	14,032,079
*Child welfare outcomes*			
Children living in deprived neighbourhoods	43.8	13.7	17.3
Children living in families on welfare	13.4	4.2	5.3

Data are presented as percentages

^a^ Individual BIG2 morbidities (combination of SGA and/or premature births) do not add up to ‘Any BIG2’ as newborns can have >1 BIG2 morbidity

^b^ = Total of foetal (from 22 weeks gestational age), intrapartum, and neonatal mortality (up to 7 days after birth)

^c^ Sum of Children 0–17 years in 2009, 2010, 2011 and 2012

In the four largest cities, considerably more mothers were of non-Western ethnicity (35% vs. 10%) and had low SES (40% vs. 16%) compared to the mothers in the rest of the Netherlands. Perinatal mortality and morbidity (i.e. BIG2) per 1000 was also higher in the four largest cities: 8.6 vs. 7.6 per 1000, and 157 vs. 139 per 1000, respectively.

The national prevalence of children living in deprived neighbourhoods and living in families on welfare were 173 and 53 per 1000 children in the Netherlands, respectively. Again, both were higher in the four largest cities; 438 vs. 137 per 1000 for children living in deprived neighbourhoods and 134 vs. 42 per 1000 for children living in families on welfare.

**Fig. 1:**
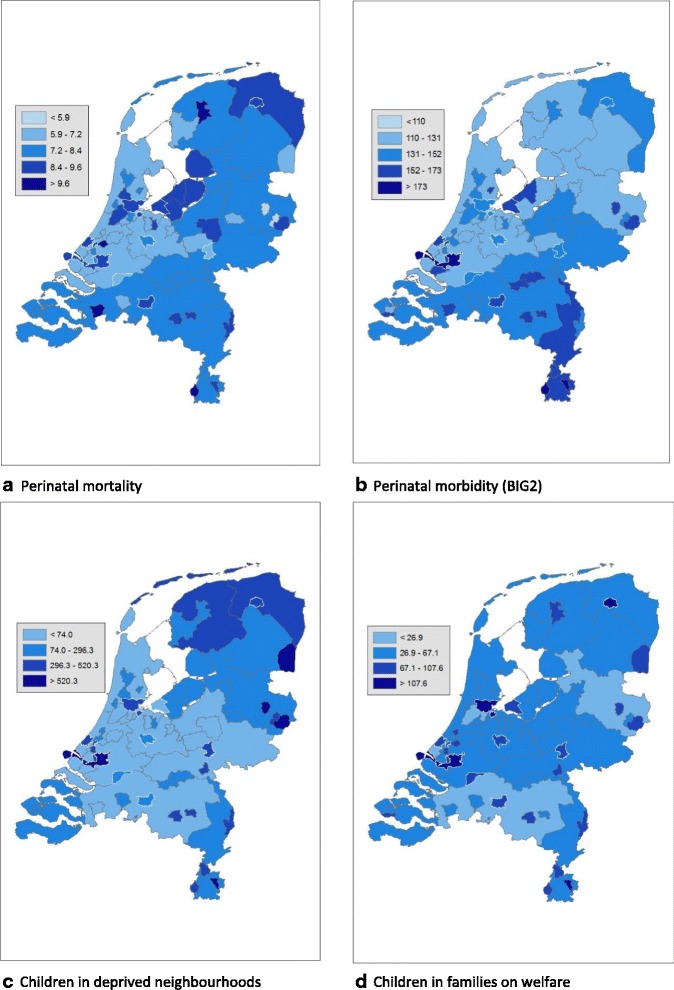
**a**-**d**. Prevalence per 1000 for 62 geographical areas in the Netherlands. The maps are based on data from Table 3, divided in five categories. The categories are formed based on the standard deviation (SD); the middle category being between −0.65 SD and 0.65 SD, the surrounding categories from plus and minus 0.65 to 1.96 SD and the outer categories below −1.96 SD and above 1.96 SD. In Fig. 1c and d the lowest category (values below −1.96 SD) does not exist due to skewedness of the data. The municipalities participating in HP4All-2 have a white border

In Table 3 the prevalence of perinatal mortality, BIG2, children living in deprived neighbourhoods, and children living in families on welfare are shown for each of the 62 geographical areas. Between geographical areas, perinatal mortality ranged from 5.3 to 10.2 per 1000 births, and perinatal morbidity ranged between 117 and 195 per 1000 births. The prevalence of children living in deprived neighbourhoods ranged between 0 and 895 per 1000, and for children living in families on welfare between 23 and 174 per 1000. The prevalence of all four outcomes in the 62 geographical areas is illustrated in Fig. 1a to d. In Additional file 1: Table S1 the prevalence of maternal age below 20 years, parity, non-Western ethnicity, and low SES tabulated for each of 62 geographical areas are presented.

**Table 3:** Prevalence (per 1000) of perinatal mortality, morbidity (BIG2), between 2009 and 2014, and children living in deprived neighbourhoods, and children living in families on welfare between 2009 and 2012, for the Netherlands and the selected 62 geographical areas

The Netherlands	Perinatal mortality	BIG2^a^	Children in deprived neighbourhoods	Children in families on welfare
	7.8	141.7	173.1	53.4
*50 largest municipalities*				
Amsterdam	8.8	151.2	450.7	144.3
Rotterdam	8.9	173.4	595.0	174.4
Den Haag	8.7	165.5	373.5	105.8
Utrecht	7.6	132.5	206.9	74.0
Eindhoven	8.8	156.5	304.1	80.8
Tilburg	8.7	170.8	246.0	78.5
Groningen	9.1	138.8	325.2	120.8
Almere	8.9	163.6	65.7	70.6
Breda	6.5	146.9	160.5	58.2
Nijmegen	7.3	145.5	337.1	93.3
Apeldoorn	8.9	136.1	35.3	43.4
Enschede	8.7	164.0	563.6	103.1
Haarlem	7.4	133.2	193.8	47.8
Arnhem	6.7	146.9	360.1	106.8
Amersfoort	6.3	127.6	35.9	45.2
Zaanstad	8.6	151.7	262.6	49.0
Den Bosch	7.8	152.5	179.4	51.7
Haarlemmermeer	8.4	133.5	0.0	24.8
Zwolle	7.3	118.2	122.2	56.4
Zoetermeer	10.2	151.8	68.6	73.1
Leiden	6.9	137.5	122.7	71.1
Maastricht	9.7	174.1	354.0	83.2
Dordrecht	7.1	146.0	261.5	71.8
Ede	6.0	117.2	0.0	37.5
Alphen a/d Rijn	6.9	120.3	10.2	36.1
Leeuwarden	9.7	136.6	291.9	98.9
Alkmaar	7.3	134.9	80.3	43.9
Emmen	6.8	145.6	650.6	68.9
Westland	7.1	121.5	2.9	23.6
Delft	8.1	144.7	308.3	95.1
Venlo	9.5	149.7	373.7	72.7
Deventer	6.8	147.8	261.7	49.4
Sittard-Geleen	7.2	160.8	384.9	72.3
Helmond	8.9	158.3	316.3	64.5
Oss	7.4	157.2	186.8	33.6
Amstelveen	7.4	139.8	0.0	25.5
Hilversum	8.9	139.4	154.5	37.3
Heerlen	9.3	195.0	895.4	124.6
Nissewaard	6.3	166.1	18.5	62.4
Sudwest Fryslan	6.7	118.2	280.0	42.2
Hengelo	5.3	137.6	380.9	56.5
Purmerend	7.5	156.0	113.8	38.1
Schiedam	8.0	167.1	328.3	101.2
Roosendaal	10.2	167.4	38.4	44.2
Lelystad	9.5	166.6	245.3	67.0
Leidschendam-Voorburg	6.5	132.5	133.6	61.2
Almelo	5.9	154.2	557.1	72.9
Hoorn	6.0	132.8	0.0	44.3
Middelburg	7.4	124.8	147.9	57.7
Vlissingen	7.4	160.2	182.2	75.5
*12 Provinces (minus 50 largest municipalities)*				
Groningen	8.9	139.0	462.2	49.8
Friesland	7.9	125.8	377.8	37.3
Drenthe	7.5	121.9	241.6	40.8
Overijssel	7.2	124.6	80.9	23.1
Gelderland	7.5	132.1	48.4	28.6
Utrecht	6.7	123.6	17.9	27.9
Noord-Holland	6.6	124.7	29.6	27.7
Zuid-Holland	7.1	131.1	55.4	32.3
Zeeland	7.7	137.6	83.9	27.2
Noord-Brabant	7.5	146.4	38.5	26.5
Limburg	8.3	159.1	136.2	44.2
Flevoland	8.8	125.6	112.1	35.0

Data are presented as promille (1 per 1000). Perinatal mortality and morbidity over the period 2009–2014 and children in deprived neighbourhoods and living in families on welfare over the period 2009–2012. Ordering of the 50 largest municipalities is based on the number of inhabitants per municipality, with the largest municipality displayed first

^a^ BIG2 combination of SGA and/or premature births

Table 4 shows the relative ranking of the ten participating municipalities in HP4All-2 for each of the four outcomes presented in Table 3.

**Table 4:** Ranking of the ten participating HP4All-2 municipalities on perinatal mortality, BIG2, children living in deprived neighbourhoods, and children living in families on welfare

	Perinatal mortality	BIG2^a^	Children in deprived neighbourhoods	Children in families on welfare
Amsterdam	15	23	7	2
Rotterdam	9	3	3	1
Den Haag	18	9	12	6
Utrecht	29	46	29	16
Tilburg	19	4	26	14
Groningen	8	36	17	4
Almere	13	11	47	23
Arnhem	52	27	13	5
Dordrecht	44	29	25	21
Schiedam	25	6	16	8

Data represent the relative ranking of the prevalence of each outcome for the ten participating HP4All-2 municipalities in the Netherlands. Rank 1 corresponds to the highest prevalence of that outcome, while rank 62 represents the lowest prevalence of that outcome

^a^BIG2 combination of SGA and/or premature births

Higher rankings correspond to higher prevalence for the corresponding outcome. Seven of the ten HP4All-2 municipalities are ranked in the top 10 for one or more of the outcomes, and all of them are placed in the top 25 for at least one of the outcomes.

## Discussion

We identified considerable variation between geographical areas within the Netherlands for perinatal mortality and morbidity, and the prevalence of children living in deprived neighbourhoods and children living in families on welfare (Table 3). This study shows that even in a high-income country such as the Netherlands, important geographical inequalities in perinatal and child health exist. The results of this study also suggest associations between adverse perinatal health and socio-economic disadvantage of children. Furthermore, when relating area-level SES (Additional file 1: Table S1) with the outcomes (Table 3) it appears that the municipalities with a higher prevalence of the study outcomes also have a higher proportion of births occurring in women from a low SES area (statistically significant positive correlation; analysis not shown). The importance of area SES and deprivation in relation to poor health outcomes in general, and more specifically perinatal and child mortality has been recognised with regards to other western countries as well [7, 15, 44, 45]. In addition to area SES and individual-level risk indicators, other area characteristics could contribute to explaining the geographical differences found in this study, such as environmental factors or population density (i.e. air pollution, minority density and distance to health care) [46–48]. Although the aim of the analyses was not to unravel the potential causes of the geographical differences, it highlights the urgency to reduce these inequalities.

The municipalities that were approached and have agreed to participate in the HP4All-2 program are among the municipalities with the most unfavourable perinatal health and/or child welfare outcomes. In the predecessor program HP4All-1, similar types of analyses were performed to identify those municipalities that had the highest rates of adverse (birth) outcomes [4]. The selection of HP4All-2 municipalities was not guided by formal analyses. Instead, selection of municipalities was guided by 1) participation in HP4All-1 and ‘Ready for a Baby’, and 2) interest shown by municipalities in the topic addressed in the program. A reason for selecting municipalities this way was that in the predecessor programs close collaboration with the participating municipalities had been established, which presumably facilitates the implementation of the HP4All-2 program studies. In these municipalities, the health care professionals, local government, and local public health services were already committed to improve perinatal outcomes via a broad multidisciplinary network [10]. Both newly selected municipalities (Dordrecht and Arnhem) have improving care for more vulnerable women and children high on the political agenda. The selection was thus merely based on effective implementation of the program in those municipalities, which we expected to have a relatively unfavourable position, not on the actual position. Nevertheless, our analyses demonstrate that most of our selected municipalities are among the worst performing in the Netherlands, with the exception of Dordrecht with a highest ranking of 21.

The intention to target high-risk municipalities with the HP4All-2 program has been based on the assumption that geographical areas with a relatively large population being at risk of adverse perinatal and child health outcomes will benefit most from interventions aimed at reducing those adverse outcomes. Sharing knowledge on how to support the most vulnerable families in the society with all involved parties is crucial, but challenging [18]. Therefore, the implementation of the HP4all-2 program, and its studies, is also expected to be challenging. Along with partnership with local parties, training sessions to share the required knowledge are being offered to health care professionals involved to help the implementation of the program.

## Conclusion

xThe ten participating municipalities in HP4All-2 all had a relatively unfavourable position regarding perinatal health and/or child welfare outcomes prior to the start of the program. In these municipalities, HP4All-2 aims to improve the care for young children and their mothers by extending the continuum for risk selection and tailored care from the preconception and prenatal period towards the postpartum, early childhood and interconception period, beyond the boundaries of separate domains of health care. By implementing and evaluating this enhanced risk management in high-risk populations, HP4All-2 aims to contribute to the reduction of (perinatal and childhood) health inequalities.

## Additional file

Additional file 1: Table S1. Demographic characteristics of the singleton pregnancies for each of the 62 selected geographical areas (per 100). (DOCX 21 kb)

## Abbreviations

HP4All: Healthy Pregnancy 4 all
PCHC: Preventive Child Health Care
R4U: Rotterdam Reproductive Risk Reduction
SES: Socioeconomic status
SGA: Small for gestational age
