# Supplementary material for: Geographical differences in perinatal health and child welfare in the Netherlands: rationale for the healthy pregnancy 4 all-2 program
Source: BMC Pregnancy Childbirth. 2017 Aug 1;17:254. doi: 10.1186/s12884-017-1425-2 (PMC5540512; doi:10.1186/s12884-017-1425-2)
Supplement: Additional file 1: Table S1. — Demographic characteristics of the singleton pregnancies for each of the 62 selected geographical areas (per 100). (DOCX 21 kb) [file 12884_2017_1425_MOESM1_ESM.docx]

**Supplementary table 1.** Demographic characteristics of the singleton pregnancies for each of the 62 selected geographical areas.

|  | **Maternal age below 20 years** | **Primiparous** | **Non-Western ethnicity** | **Low SES** |
| --- | --- | --- | --- | --- |
| The Netherlands | 1.3 | 45.8 | 14.2 | 20.0 |
| ***50 largest municipalities*** | | | | |
| Amsterdam | 1.3 | 50.0 | 35.5 | 38.2 |
| Rotterdam | 2.5 | 48.6 | 38.2 | 57.41 |
| Den Haag | 1.7 | 47.1 | 36.9 | 37.04 |
| Utrecht | 0.6 | 49.8 | 20.9 | 17.22 |
| Eindhoven | 1.2 | 48.9 | 20.9 | 20.81 |
| Tilburg | 1.6 | 48.6 | 17.9 | 36.49 |
| Groningen | 1.6 | 51.9 | 9.9 | 39.3 |
| Almere | 2.1 | 44.2 | 34.5 | 4.74 |
| Breda | 1.5 | 48.5 | 12.9 | 21.08 |
| Nijmegen | 1.1 | 49.5 | 11.4 | 28.45 |
| Apeldoorn | 1.4 | 45.6 | 9.6 | 9.52 |
| Enschede | 2.2 | 46.8 | 15.2 | 56.99 |
| Haarlem | 0.9 | 49.4 | 12.7 | 16.09 |
| Arnhem | 1.2 | 49.2 | 18.6 | 41.91 |
| Amersfoort | 1.0 | 44.3 | 13.7 | 12.14 |
| Zaanstad | 1.2 | 44.6 | 23.6 | 20.06 |
| Den Bosch | 0.9 | 49.3 | 11.9 | 29.53 |
| Haarlemmermeer | 0.5 | 44.1 | 13.1 | 0 |
| Zwolle | 1.4 | 45.9 | 7.3 | 19.17 |
| Zoetermeer | 1.8 | 45.6 | 18.9 | 6.65 |
| Leiden | 0.9 | 49.6 | 15.2 | 9.82 |
| Maastricht | 2.4 | 50.5 | 11.8 | 38.44 |
| Dordrecht | 2.0 | 46.7 | 18.4 | 39.32 |
| Ede | 1.6 | 40.4 | 7.8 | 0 |
| Alphen a/d Rijn | 0.7 | 45.7 | 10.7 | 0 |
| Leeuwarden | 2.2 | 49.0 | 10.5 | 33.2 |
| Alkmaar | 1.3 | 48.6 | 15.2 | 9.65 |
| Emmen | 2.1 | 45.9 | 6.6 | 67.88 |
| Westland | 0.7 | 43.7 | 5.4 | 0.42 |
| Delft | 1.6 | 49.5 | 21.0 | 14.65 |
| Venlo | 1.4 | 46.3 | 14.0 | 39.69 |
| Deventer | 1.4 | 46.3 | 10.2 | 32.65 |
| Sittard-Geleen | 1.8 | 49.0 | 8.8 | 50.08 |
| Helmond | 1.3 | 45.8 | 13.1 | 38.31 |
| Oss | 1.0 | 48.1 | 11.1 | 14.02 |
| Amstelveen | 0.3 | 45.1 | 22.9 | 0 |
| Hilversum | 1.3 | 48.1 | 14.4 | 4.26 |
| Heerlen | 2.9 | 50.2 | 11.0 | 88.62 |
| Nissewaard | 2.1 | 48.5 | 12.2 | 0 |
| Sudwest Fryslan | 1.4 | 41.9 | 2.8 | 31.75 |
| Hengelo | 1.5 | 46.3 | 10.7 | 42.74 |
| Purmerend | 1.3 | 48.2 | 16.1 | 8.78 |
| Schiedam | 2.1 | 47.1 | 37.3 | 34.81 |
| Roosendaal | 1.5 | 55.2 | 16.4 | 54.67 |
| Lelystad | 2.6 | 41.6 | 21.1 | 25.57 |
| Leidschendam-Voorburg | 1.1 | 48.5 | 16.1 | 8.86 |
| Almelo | 2.1 | 44.3 | 12.8 | 69.55 |
| Hoorn | 1.3 | 48.7 | 14.4 | 12.15 |
| Middelburg | 1.4 | 42.7 | 9.8 | 16.9 |
| Vlissingen | 2.2 | 46.2 | 9.7 | 19.0 |
| ***12 Provinces (minus 50 largest municipalities)*** | | | | |
| 1. Groningen | 2.0 | 44.0 | 5.6 | 46.9 |
| 1. Friesland | 1.4 | 42.7 | 3.9 | 35.0 |
| 1. Drenthe | 1.4 | 41.6 | 4.0 | 21.0 |
| 1. Overijssel | 0.8 | 41.9 | 2.9 | 5.3 |
| 1. Gelderland | 1.2 | 44.1 | 5.6 | 7.0 |
| 1. Utrecht | 0.9 | 42.9 | 9.6 | 2.6 |
| 1. Noord-Holland | 1.0 | 44.3 | 8.4 | 2.1 |
| 1. Zuid-Holland | 1.0 | 43.8 | 10.9 | 5.2 |
| 1. Zeeland | 1.5 | 42.3 | 4.8 | 2.3 |
| 1. Noord-Brabant | 0.8 | 45.8 | 6.2 | 4.9 |
| 1. Limburg | 1.2 | 48.1 | 6.9 | 17.2 |
| 1. Flevoland | 1.6 | 41.1 | 3.9 | 2.2 |

Data are presented as percentiles (1 per 100, %). Ordering of the 50 largest municipalities is based on the number of inhabitants per municipality, with the largest municipality displayed first.
